# Multiscale cardiorespiratory complexity reveals autonomic signatures of Rajyoga meditation

**DOI:** 10.3389/fpsyg.2026.1792917

**Published:** 2026-06-04

**Authors:** Raghuwansh Singh, Rajanikant Panda, Mika P. Tarvainen, Vivek Ranjan, Anindita Ganguly, Suman Halder

**Affiliations:** 1 Department of Electrical Engineering, National Institute of Technology Durgapur, Durgapur, West Bengal, India; 2 Department of Neurology, University of California, San Francisco, San Francisco, CA, United States; 3 Department of Technical Physics, University of Eastern Finland, Kuopio, Finland; 4 Directorate of Technical Education, Higher Education, Government of West Bengal, Kolkata, West Bengal, India

**Keywords:** autonomic nervous system, heart rate variability, multiscale entropy, Rajyoga meditation, respiration

## Abstract

**Introduction:**

Meditation offers a tractable model for probing altered states of consciousness, yet consistent physiological markers remain elusive. This study characterized within-subject autonomic and respiratory modulation across pre-meditation, during-meditation, and post-meditation states in trained Rajyoga practitioners using multiscale heart rate variability (HRV) metrics and HRV–respiration coupling. Sex-stratified analyses were presented exploratorily to summarize state-dependent effects.

**Methods:**

A single-lead electrocardiogram (ECG) was recorded from 55 Rajyoga practitioners (31 male, 24 female) during three consecutive 10-min states: pre-meditation, during meditation, and post-meditation. After artifact correction, 27 HRV indices spanning time-domain, frequency-domain, and non-linear dynamics, including multiscale entropy (MSE 1–20), were computed in Kubios HRV Scientific. State-dependent effects were tested using Friedman tests with Bonferroni-adjusted post-hoc contrasts, and cardiorespiratory coupling was assessed through correlations between respiration frequency and low-frequency (LF) and high-frequency (HF) HRV powers.

**Results:**

Meditation was associated with robust state-dependent modulation. Time-domain indices and frequency-domain powers increased during meditation with partial post-session recovery, while selected non-linear features shifted significantly. Respiration frequency was reduced, and HRV–respiration coupling was strengthened, consistent with increased parasympathetic engagement. Exploratory sex-stratified summaries indicated potential differences in effect magnitudes that warrant confirmation in studies powered for interaction testing.

**Discussion:**

These findings identify a reproducible physiological signature of the meditative state by integrating HRV magnitude, complexity, and cardiorespiratory coupling. The results support the usefulness of multiscale cardiorespiratory analysis as an operational marker of altered consciousness induced by Rajyoga meditation.

## 1 Introduction

Meditation has been conceptualized as a family of attentional and emotional regulatory practices through which wellbeing, emotional balance, and altered states of consciousness may be cultivated (Lutz et al., 2008). In psychological and behavioral science, meditation is therefore studied not only as a spiritual discipline but also as a form of mental training relevant to self-regulation, and mind-body functioning (Lutz et al., 2008; Panda et al., 2016). Across clinical and non-clinical samples, different meditation practice have shown small to moderate benefits for anxiety, depression, and related stress outcomes (Vishnubhotla et al., 2024), which supports continued interest in the psychological mechanisms through which meditation may influence health and behavior (Lutz et al., 2008) Rajyoga meditation is a useful practice to examine within this broader literature because it is commonly practised with open eyes and is intended to be integrated into ordinary daily life rather than restricted to formal, eyes-closed sessions. Studies have shown that, Rajyoga practitioners have greater positive thinking and happiness, reduced anxiety and cortisol in stressful clinical settings, and differences in brain systems involved in self-related thought and reward processing. Although there is limited evidence on clinical application, it suggests that Rajyoga may influence both psychological functioning and autonomic physiology, making it relevant for psychophysiological research (Mehta et al., 2020; Babu et al., 2020; Aggarwal et al., 2017).

One way to study these links is through heart rate variability (HRV), the natural variation in the time interval between successive heartbeats. HRV has been widely used as a non-invasive index of autonomic and cardiovascular regulation (Task Force of the European Society of Cardiology and the North American Society of Pacing and Electrophysiology, 1996; Laborde et al., 2017). Rather than representing “noise” in cardiac timing, HRV has been understood as a marker of the heart's adaptive flexibility in response to internal and external demands (Laborde et al., 2017). In psychophysiology, HRV has been linked with cardiac vagal control and has been associated with processes that are highly relevant to psychology, including emotion regulation, cognitive control, social engagement, stress responsivity, and broader self regulatory capacity (Holzman and Bridgett, 2017). For this reason, HRV has been considered useful in meditation research, where changes in breathing, attention, and affective state are expected to interact with autonomic control. At the same time, it has been emphasized in recent reporting guidelines that HRV must be interpreted carefully, because recording conditions, pre-processing choices, respiratory influences, and metric selection can all affect conclusions (Laborde et al., 2017; Quigley et al., 2024).

In the context of meditation, HRV is therefore useful because it offers a physiological window into whether shifts in attention, affect, and stress are accompanied by measurable changes in autonomic regulation. Respiratory sinus arrhythmia and related high frequency fluctuations in heart period are strongly shaped by breathing rate and depth, so changes observed during meditation cannot be interpreted adequately without attention to respiration and cardiorespiratory coupling (Grossman, 2024; Dick et al., 2014). In addition, not all HRV indices provide the same information. Conventional time domain and frequency domain measures remain informative, but non-linear measures have increasingly been used to characterize complexity, irregularity, and multiscale organization in physiological time series that may not be visible in simpler summary metrics (Costa et al., 2002). At the same time, simplified interpretations of the ratio of low frequency to high frequency power (LF/HF) as a direct measure of “sympathovagal balance” have been questioned, and a more cautious, physiology informed interpretation has been recommended (Quigley et al., 2024; Grossman, 2024). Taken together, these considerations indicate that meditation-related autonomic change is better understood when HRV magnitude, respiratory modulation, and non-linear complexity are examined jointly rather than in isolation.

A substantial body of literature has suggested that meditation and mindfulness practices may alter autonomic function, often in a direction consistent with increased parasympathetic engagement or improved regulatory flexibility (Laborde et al., 2017; Brown et al., 2021; Natarajan, 2023). However, the evidence has remained heterogeneous. In a meta-analysis of randomized controlled trials, clear improvements in vagally mediated resting-state HRV were not observed consistently across mindfulness and meditation interventions, and the need for larger, methodologically stronger studies was emphasized (Brown et al., 2021). This heterogeneity has likely arisen from differences in meditation style, participant experience, recording duration, respiratory handling, outcome selection, and statistical reporting (Quigley et al., 2024; Brown et al., 2021). These issues are particularly relevant in practices in which breathing rhythm itself may be altered, because apparent autonomic effects may partly reflect respiratory patterning rather than a unitary shift in autonomic state (Grossman, 2024; Natarajan, 2023). Thus, although meditation related HRV modulation has often been reported, the literature has not yet provided a fully consistent picture.

The need for greater clarity is also evident in Rajyoga specific research. Rajyoga meditation has been less frequently studied than several other contemplative traditions, despite evidence suggesting that it may influence both neural and autonomic processes (Panda et al., 2016; Babu et al., 2020; Jha et al., 2025). Existing Rajyoga studies have reported altered default-mode-network dynamics, structural differences in brain regions related to reward and positive affect, and improved HRV in clinical intervention settings (Babu et al., 2020; Jha et al., 2025). Yet several gaps have remained. First, much of the broader meditation-HRV literature has focused on only two states, most commonly pre-meditation and during meditation, or baseline and post-intervention comparisons, whereas within-session recovery after meditation has been less thoroughly characterized (Brown et al., 2021; Natarajan, 2023; Nasrolahzadeh et al., 2024). Second, many studies have relied mainly on conventional HRV indices, whereas integrated analyses combining time-domain, frequency-domain, non-linear, and respiration related measures have been less common (Grossman, 2024; Costa et al., 2002; Natarajan, 2023; Nasrolahzadeh et al., 2024). Third, open eye Rajyoga practice in trained practitioners has remained comparatively underrepresented in the HRV literature. Finally, sex stratified descriptions of state dependent physiological modulation have been limited, even though sex related variation in autonomic regulation may influence the observed pattern of effects.

Against this background, the present study examined state dependent autonomic and cardiorespiratory changes across pre-meditation, meditation, and post-meditation periods in trained Rajyoga practitioners. Using electrocardiogram (ECG) derived HRV together with respiration-related measures, the study assessed standard HRV indices, complexity-based indices, and the coordination between breathing and heart rate (HR) fluctuations across the three states. This approach was intended to characterize the physiological signature of the meditative state and to describe immediate post-meditation recovery. Because sex differences in cardiac autonomic regulation have been reported in healthy adults, male and female subgroups were also described separately as exploratory analyses. It was hypothesized that Rajyoga meditation would be associated with slower breathing, greater parasympathetic-linked HRV during practice, altered heart–breath coordination, and partial return toward baseline after meditation.

## 2 Materials and methods

This section describes the study design, participant enrolment, sample size justification, experimental protocol, signal acquisition and pre-processing, HRV feature extraction, statistical analysis, and cardiorespiratory correlation analysis.

### 2.1 Study design

This study was designed as a single center, observational, within subject repeated-measures investigation. Each participant underwent one continuous 30-min ECG recording session composed of three consecutive 10-min physiological states: pre-meditation rest, during Rajyoga meditation, and post-meditation recovery. The same seated posture, recording instrument, and acquisition settings were maintained throughout the session for each participant. The primary inferential comparison was defined as the within-subject change across the three repeated states. Additional sex stratified analyses were conducted separately in male and female subgroups and were interpreted as exploratory subgroup summaries rather than confirmatory interaction tests. The study was performed at the National Institute of Technology Durgapur, West Bengal, India, in collaboration with the Brahma Kumaris Rajyoga Meditation Center, India. Written informed consent was obtained from all participants before enrolment, and ethical approval was obtained from the Institutional Ethics Committee (reference number: NITD/IEC/3-25), and the study was conducted in accordance with the ethical principles of the Declaration of Helsinki.

### 2.2 Subject enrolment

A total of 64 participants were enrolled from the Brahma Kumaris Rajyoga Meditation Center. Eligibility required at least 1 year of regular Rajyoga practice and at least 1 h of daily meditation. Participants with cardiovascular, respiratory, or neurological disorders were excluded. Nine recordings were excluded because of technical interruption or electrode displacement, leaving 55 participants for final analysis. Table 1 presents all the demographic characteristics of the registered Rajyoga practitioners.

**Table 1:** Demographic and anthropometric characteristics of the analyzed Rajyoga practitioners.

Characteristic	Male (*n* = 31)	Female (*n* = 24)
Sex, n (%)	31 (56.4%)	24 (43.6%)
Age, years (mean ± SD)	40.87 ± 16.90	34.75 ± 15.89
Weight, kg (mean ± SD)	66.90 ± 11.87	58.08 ± 11.11
Height, cm (mean ± SD)	170.81 ± 5.28	157.12 ± 12.01
BMI, kg/m^2^ (mean ± SD)	22.87 ± 3.58	23.65 ± 4.53

Values are reported as mean ± standard deviation unless otherwise stated. BMI, body mass index; SD, standard deviation.

### 2.3 Sample size justification

The sample size was justified by a sensitivity based analysis. The sensitivity analysis was performed separately for the male and female subgroups. The achieved subgroup sample sizes were therefore evaluated for the three conditions of pre meditation, during meditation, and post-meditation, and for the same omnibus inferential procedure, namely the Friedman test.

For sample-size justification, standard deviation of normal normal intervals (SDNN) and root mean square of successive differences (RMSSD) were retained as the primary outcomes in the main sensitivity findings because these variables were the most interpretable and yielded the most favorable sensitivity profile in both subgroup analyses. Sensitivity was quantified in terms of the minimum detectable standardized within-subject effect at α = 0.05 and target power = 0.80. Simulation-based evaluation was used because it is well-suited to repeated measures settings in which direct closed form solutions may be less convenient, and bootstrap based interval estimation is an established approach for effect-size uncertainty reporting.

In the male subgroup (*n* = 31), the minimum detectable standardized effect was 0.15 for both SDNN and RMSSD at α = 0.05 and 80% power. Within the evaluated sample size grid for a moderate standardized effect (δ = 0.50), 10 participants were sufficient to achieve 80% power, indicating that the achieved male sample size was well above that threshold. While in the female subgroup (*n* = 24), the minimum detectable standardized effect was 0.20 for both SDNN and RMSSD at α = 0.05 and 80% power. Within the evaluated sample-size grid for a moderate standardized effect (δ = 0.50), 10 participants were sufficient to achieve 80% power, again indicating that the achieved female sample size exceeded that level. Table 2 shows the sample size estimation based on the standardized delta (MDE).

**Table 2:** Power analysis parameters for sample size estimation based on the standardized delta (MDE).

Group	Feature	Subjects	MDE standardized	Required n for	Alpha	Target
			delta	delta = 0.50		power
Male	SDNN	31	0.15	10	0.05	0.80
Male	RMSSD	31	0.15	10	0.05	0.80
Female	SDNN	24	0.20	10	0.05	0.80
Female	RMSSD	24	0.20	10	0.05	0.80

The table reports the minimum detectable standardized within subject effect, required sample size for a moderate effect, achieved subgroup size, target power, and alpha level for the repeated-measures design used in the study. MDE, minimum detectable effect.

Previous meditation-based repeated measures work by Peng et al. (2004) examined 10 experienced meditators and reported significant changes in HR dynamics across meditation conditions; however, in the present study, sample adequacy has not been inferred from precedent alone and has instead been supported by an explicit sensitivity based justification.

### 2.4 Experimental procedures

**Figure 1:**
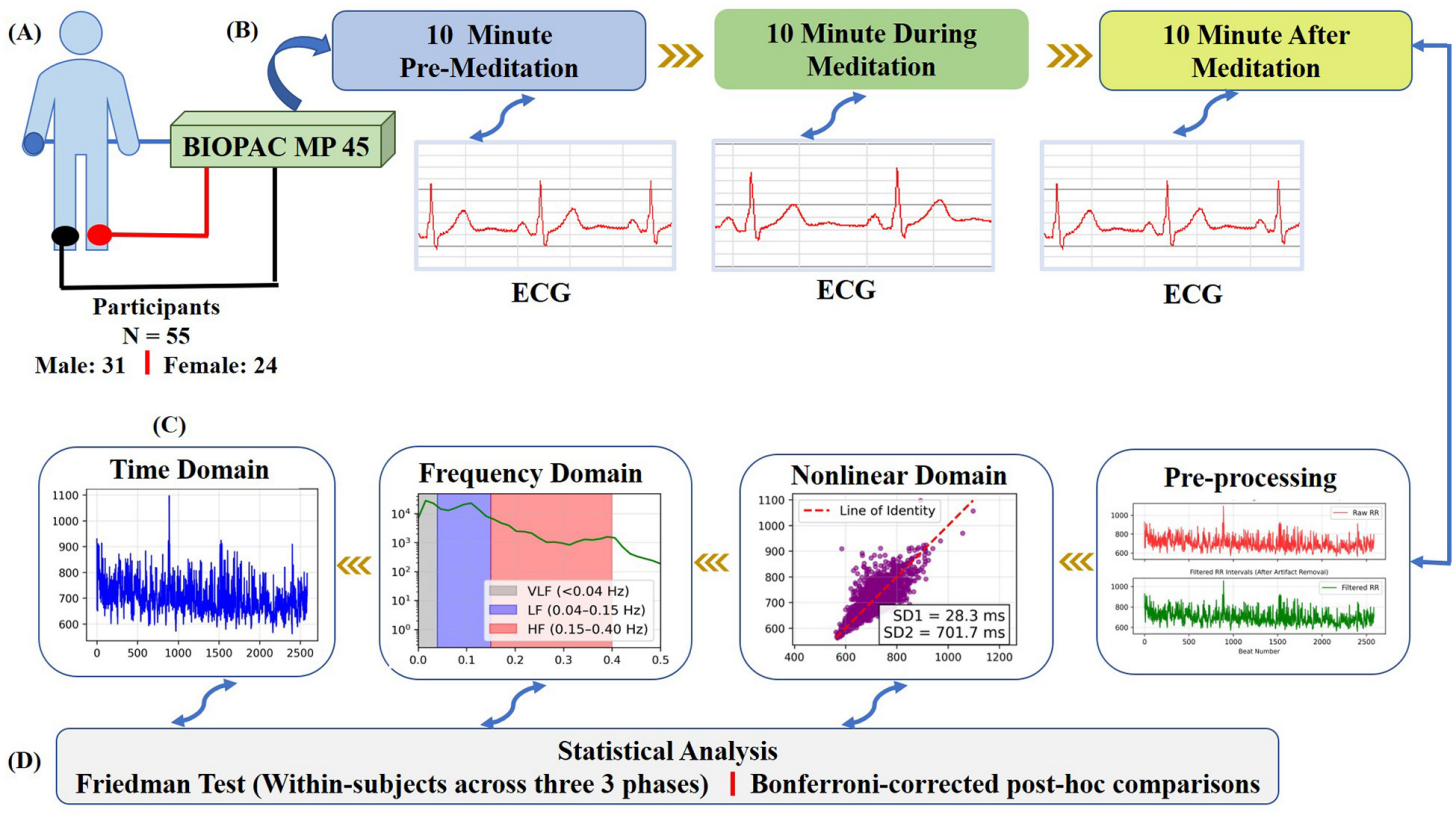
Overview of the proposed experimental design and analysis pipeline. **(A)** Illustration of ECG electrode placement and recording setup. **(B)** Experimental design showing the three recording conditions: 10 min of pre-meditation, followed by 10 min of meditation and 10 min of post-meditation. In all three conditions, ECGs were recorded. **(C)** ECG feature extraction and analysis across the time domain, frequency domain, and non-linear domain measures. **(D)** Statistical analysis workflow.

After electrode placement, the participants were seated comfortably on a chair in a quiet recording setting, and a continuous 30-min ECG recording session was performed under a fixed acquisition configuration. The recording protocol was composed of three consecutive, non-overlapping 10-min states. In the pre-meditation state, the participants were instructed to remain awake, relaxed, and with open eyes, without intentionally engaging in meditation or directed mental imagery. This was followed immediately by a 10 min during meditation state, in which open eye Rajyoga meditation was practiced using the Soul Consciousness method, a classical Rajyoga technique characterized by disengagement from external awareness and sustained focused attention toward positive self reflective virtues (Panda et al., 2016; Kumar et al., 2024). In the final 10 min post-meditation state, meditation was discontinued and the participants were instructed to remain seated, awake, relaxed, and with open eyes. No interruption was introduced between the three segments, and the full 30 min recording was subsequently segmented into three fixed 10 min epochs corresponding to pre-meditation, during meditation, and post-meditation. The experimental sequence is illustrated in Figure 1.

### 2.5 Data acquisition and pre-processing

Continuous single-lead ECG was acquired using the BIOPAC MP45 system (BIOPAC Systems, Inc., Goleta, CA, USA) at a sampling frequency of 2,000 Hz. After cleaning the skin surface with alcohol, three Ag/AgCl electrodes were placed in a Lead II configuration. The SS2LB lead wire was connected to the right leg (positive terminal), left leg (ground), and right hand (negative terminal) (Kumar et al., 2023). The raw ECG recordings were visually screened for technical interruptions and gross motion-related disturbance. Power-line interference at 50 Hz was attenuated using a notch filter (Gupta et al., 2024). Baseline wander was attenuated using a filter with a passband of 0.05–100 Hz (Mondal et al., 2024). Ectopic beats and abnormal intervals were detected and corrected in Kubios HRV Scientific using the same pre-processing pipeline for all participants. Each 30-min recording was then divided into three non-overlapping 10-min epochs corresponding to pre meditation, during meditation, and post-meditation. Recordings affected by major technical interruption or electrode displacement were excluded before statistical analysis. Table 3 shows the acquired ECG signals in the three state Rajyoga meditation protocol.

**Table 3:** Acquisition characteristics of the ECG epochs used in the three state Rajyoga meditation protocol.

Dataset	No. of subject	ECG signal	Sampling	Time (sec)
		length	frequency (HZ)	
Pre-meditation	55	1,200,000	2,000	600
During meditation	55	1,200,000	2,000	600
Post meditation	55	1,200,000	2,000	600

Each state corresponded to a 10 min ECG segment acquired at 2,000 Hz. ECG signal length of 1,200,000 denotes the number of samples in each 10 min epoch, calculated as 2,000 Hz × 600 s.

### 2.6 HRV feature extraction

HRV analysis was performed from artifact corrected normal to normal interval after exclusion of artifacts (NN) interval series using the same software pipeline for all recordings. The extracted features were grouped into time, frequency, and non-linear indices. The time domain measures included Mean interval between two successive R peaks (RR), Mean HR, SDNN, RMSSD, standard deviation of heart rate (STD HR), number of successive normal to normal interval pairs differing by more than 50 ms (NN50), HRV triangular index (HRV TI), and triangular interpolation of normal-to-normal interval histogram (TINN). The frequency domain measures included low-frequency power (LF), high-frequency power (HF), and LF/HF ratio derived from spectral analysis. Non-linear measures included Poincare plot, entropy based indices, detrended fluctuation analysis, recurrence plot measures, correlation dimension, and multiscale entropy across scales 1–20. Respiration frequency was estimated from ECG derived respiration within the same analytical environment. The mathematical definitions of the principal measures used in the study are presented in Equations 1–16.

The mean RR interval $\bar{\text{RR}}$ and mean HR $\bar{\text{HR}}$ are calculated for an N-point beat to beat RR interval time series (RR = (RR_1_, RR_2_, RR_3_, …, RR_*N*_)). Mean RR and Mean HR were calculated using Equations 1 and 2.


$$\begin{array}{c} \bar{\text{RR}}=\frac{1}{N}\overset{N}{\underset{j=1}{\sum }}\text{R}{\text{R}}_{j} \end{array}$$ (1)

$$\begin{array}{c} \bar{\text{HR}}=\frac{1}{N}\overset{N}{\underset{j=1}{\sum }}\frac{60}{\text{R}{\text{R}}_{j}} \end{array}$$ (2)

The mean values, along with the SDNN and RMSSD, are used to evaluate the variability in the beat to beat RR interval values. These measures determine how much the RR intervals differ from one another. SDNN and RMSSD were calculated using Equations 3 and 4.


$$\begin{array}{c} \text{SDNN}=\sqrt{\frac{1}{N-1}\overset{N}{\underset{j=1}{\sum }}{\left(\text{R}{\text{R}}_{j}-\bar{\text{RR}}\right)}^{2}} \end{array}$$ (3)

$$\begin{array}{c} \text{RMSSD}=\sqrt{\frac{1}{N-1}\overset{N-1}{\underset{j=1}{\sum }}{\left(\text{R}{\text{R}}_{j+1}-\text{R}{\text{R}}_{j}\right)}^{2}} \end{array}$$ (4)

NN50 is an HRV metric that measures the number of consecutive RR intervals that have a difference greater than 50 ms. pNN50 is a commonly used parameter that represents a percentage value. pNN50 was calculated using Equation 5.


$$\begin{array}{c} p\text{NN}50=\frac{\text{NN}50}{N-1}\times 100\% \end{array}$$ (5)

HRV frequency domain measures are evaluated using power spectral density (PSD) analysis to examine the HRV time series in the frequency domain. Two methods are employed to estimate the spectrum of the RR interval time series: Welch's periodogram and AR spectrum estimation. In this study, Welch's periodogram technique was used for the spectrum estimation. The relative HRV power of the LF and HF bands can be expressed in normalized units (n.u.). Frequency-domain powers and normalized/relative powers were calculated using Equations 6–10, and the LF/HF ratio was calculated using Equation 11.


$$\begin{array}{c} P_{\text{LF}}\left(\text{n}.\text{u}.\right)=\frac{P_{\text{LF}}\left(\text{m}{\text{s}}^{2}\right)}{P_{\text{Total}}\left(\text{m}{\text{s}}^{2}\right)-P_{\text{VLF}}\left(\text{m}{\text{s}}^{2}\right)}\times 100\% \end{array}$$ (6)

$$\begin{array}{c} P_{\text{HF}}\left(\text{n}.\text{u}.\right)=\frac{P_{\text{HF}}\left(\text{m}{\text{s}}^{2}\right)}{P_{\text{Total}}\left(\text{m}{\text{s}}^{2}\right)-P_{\text{VLF}}\left(\text{m}{\text{s}}^{2}\right)}\times 100\% \end{array}$$ (7)

while percentage of the relative HRV power of the VLF, LF, and HF bands is as follows:


$$\begin{array}{c} P_{\text{VLF}}\left(\%\right)=\frac{P_{\text{VLF}}\left(\text{m}{\text{s}}^{2}\right)}{P_{\text{Total}}\left(\text{m}{\text{s}}^{2}\right)}\times 100\% \end{array}$$ (8)

$$\begin{array}{c} P_{\text{LF}}\left(\%\right)=\frac{P_{\text{LF}}\left(\text{m}{\text{s}}^{2}\right)}{P_{\text{Total}}\left(\text{m}{\text{s}}^{2}\right)}\times 100\% \end{array}$$ (9)

$$\begin{array}{c} P_{\text{HF}}\left(\%\right)=\frac{P_{\text{HF}}\left(\text{m}{\text{s}}^{2}\right)}{P_{\text{Total}}\left(\text{m}{\text{s}}^{2}\right)}\times 100\% \end{array}$$ (10)

The power ratio of LF and HF is calculated by


$$\begin{array}{c} \frac{\text{LF}}{\text{HF}}=\frac{P_{\text{LF}}\left(\text{m}{\text{s}}^{2}\right)}{P_{\text{HF}}\left(\text{m}{\text{s}}^{2}\right)} \end{array}$$ (11)

When examining the proportion of LF to HF, the relative powers of different frequency components and the LF/HF ratio are useful, as they are known to reflect sympatho-vagal balance. The Poincaré plot, which plots RR_*j*+1_ as a function of RR_*j*_, is a graphical depiction that shows the association between consecutive RR intervals. The plot's form is determined by fitting an ellipse to the data points (RR_*j*_, RR_*j*+1_) aligned with the line of identity (LOI), where RR_*j*_ = RR_*j*+1_. SD1 represents the standard deviation that is perpendicular to the LOI, whereas SD2 represents the standard deviation that is along the LOI. Poincaré plot descriptors SD1 and SD2 were calculated using Equations 12 and 13. Approximate entropy and sample entropy were calculated using Equations 14 and 15, and DFA fluctuation was calculated using Equation 16.


$$\begin{array}{c} \text{SD}1=\sqrt{\frac{\text{RMSS}{\text{D}}^{2}}{2}} \end{array}$$ (12)

$$\begin{array}{c} \text{SD}2=\sqrt{2\text{SDN}{\text{N}}^{2}-\frac{\text{RMSS}{\text{D}}^{2}}{2}} \end{array}$$ (13)

Approximate Entropy (ApEn) measures the complexity and irregularity of HR dynamics. Sample Entropy (SampEn) is an improved version of Approximate Entropy that provides a more consistent and less biased measure of complexity and irregularity in time series data.


$$\begin{array}{c} \text{ApEn}\left(m,r,n\right)={\text{Ψ}}^{m}\left(r\right)-{\text{Ψ}}^{m+1}\left(r\right) \end{array}$$ (14)

$${\text{Ψ}}^{m}\left(r\right)=\frac{1}{N-m+1}\overset{N-m+1}{\underset{j=1}{\sum }}C_{j}^{m}\left(r\right)$$

$$\begin{array}{c} \text{SampEn}\left(m,r,N\right)=\ln\left(\frac{{\text{Ψ}}^{m}\left(r\right)}{{\text{Ψ}}^{m+1}\left(r\right)}\right) \end{array}$$ (15)

N is the number of beats, r is the tolerance value, and m is the embedding dimension in both SampEn and ApEn. HR time series correlation characteristics are correlated by Detrended Fluctuation Analysis (DFA). For every section, the root mean square fluctuation of the integrated and detrended time series is computed using the fluctuation function.


$$\begin{array}{c} F\left(n\right)=\sqrt{\frac{1}{N}\overset{N}{\underset{i=1}{\sum }}{\left[y\left(i\right)-y_{n}\left(i\right)\right]}^{2}} \end{array}$$ (16)

Where $y\left(x\right)=\overset{x}{\underset{i=1}{\sum }}\left(\text{R}{\text{R}}_{i}-\bar{\text{RR}}\right)$. The slopes of the log-log plot of the fluctuation function *F*(*n*) against segment length n in the corresponding short and long term areas are α_1_ and α_2_.

Recurrence plot (RP) based features were also extracted. As RP and the associated recurrence quantification analysis measures included DET (determinism), Lmean (mean diagonal line length), and ShanEn (Shannon entropy of the diagonal line length distribution). The correlation dimension (CD) D2 was also calculated.

### 2.7 Statistical analysis

Continuous variables were summarized using the median and interquartile range (25^th^ and 75^th^). Because the repeated-measures data were not normally distributed, within subject differences across pre meditation, during meditation, and post-meditation were evaluated using the Friedman test. When the omnibus Friedman test was significant, pairwise *post-hoc* comparisons were performed using Bonferroni correction, and the corrected threshold was set at α_corrected_ = 0.05/3 = 0.0167. The pre-specified primary outcomes were SDNN and RMSSD. The remaining HRV variables, multiscale entropy measures, and correlation analyses were treated as secondary or exploratory outcomes. Analyses were performed in the full cohort and separately in male and female subgroups. For the primary outcomes, effect magnitude was summarized using Kendall's W with bootstrap 95% confidence intervals. The analytical workflow was implemented in Python, and feature extraction was performed in Kubios HRV Scientific (Tarvainen et al., 2014).

### 2.8 Cardiorespiratory correlation analysis

Cardiorespiratory coupling was evaluated across the pre meditation, during meditation, and post-meditation states using state wise correlations between respiration frequency and LF power, and between respiration frequency and HF power. Correlations between LF power and HF power were also summarized. Correlation analysis was performed separately for the male and female subgroups to describe state-dependent coupling patterns within each subgroup. Correlations were calculated for both groups individually, providing a detailed comparison of these relationships within and across the different phases of meditation. A respiratory belt was not used in the present protocol because the recording session was designed as a minimally instrumented ECG based acquisition in order to reduce instrumentation burden during the meditation task. Respiration was therefore estimated from the ECG using the ECG derived respiration function implemented in Kubios HRV (Tarvainen et al., 2014).

## 3 Results

Overall, 27 features in males and 22 features in females showed significant state dependent modulation, consistent with a shift toward parasympathetic predominance during Rajyoga meditation.

### 3.1 Dynamic change in autonomic nervous system (ANS)

#### 3.1.1 Time and frequency domain

Changes in HRV parameters from ECG recordings of 55 human subjects were used to investigate the impact of RM on the activity of the ANS. The pre meditation, meditation, and post-meditation phases were each segmented into a 10 min interval, and the analysis was conducted over a 30 min recording.

**Figure 2:**
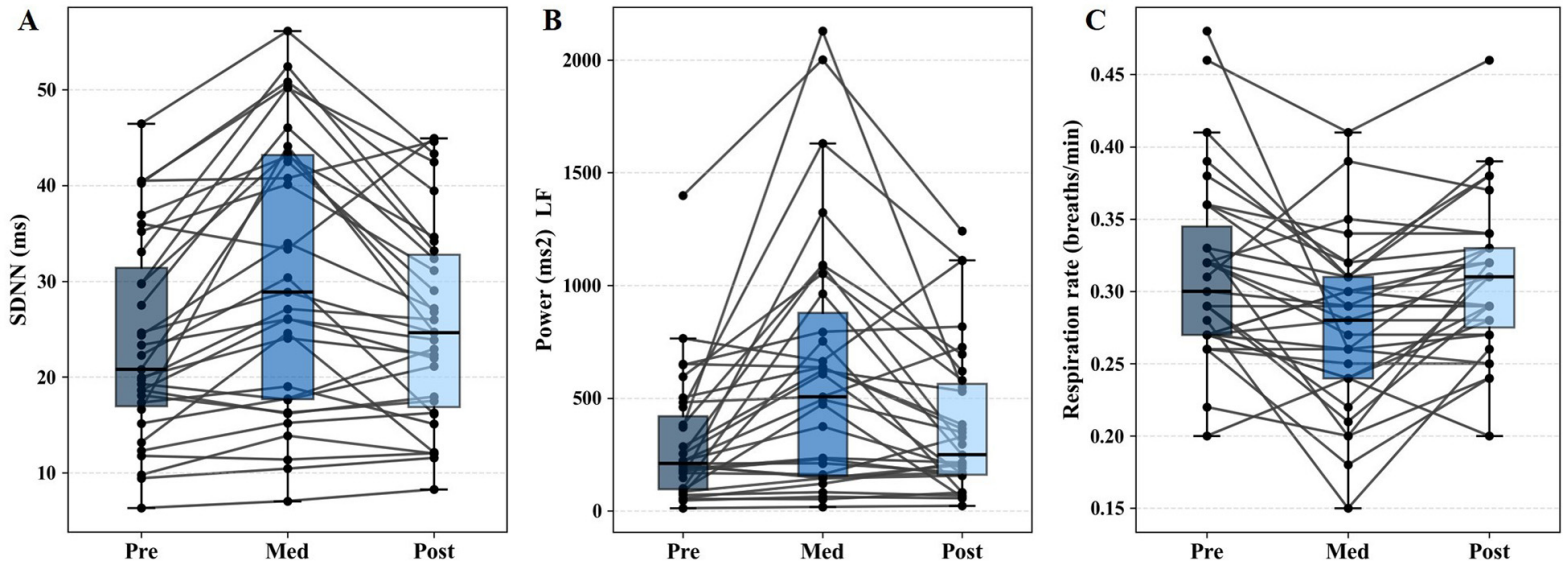
Male linear HRV and respiration-frequency changes across the three physiological states. Boxplots of all subjects' trajectories are shown for representative linear indices in the male group across pre-meditation, during meditation, and post-meditation. **(A)** SDNN, **(B)** LF power, and **(C)** respiration frequency. SDNN, standard deviation of NN intervals; LF, low-frequency power.

**Figure 3:**
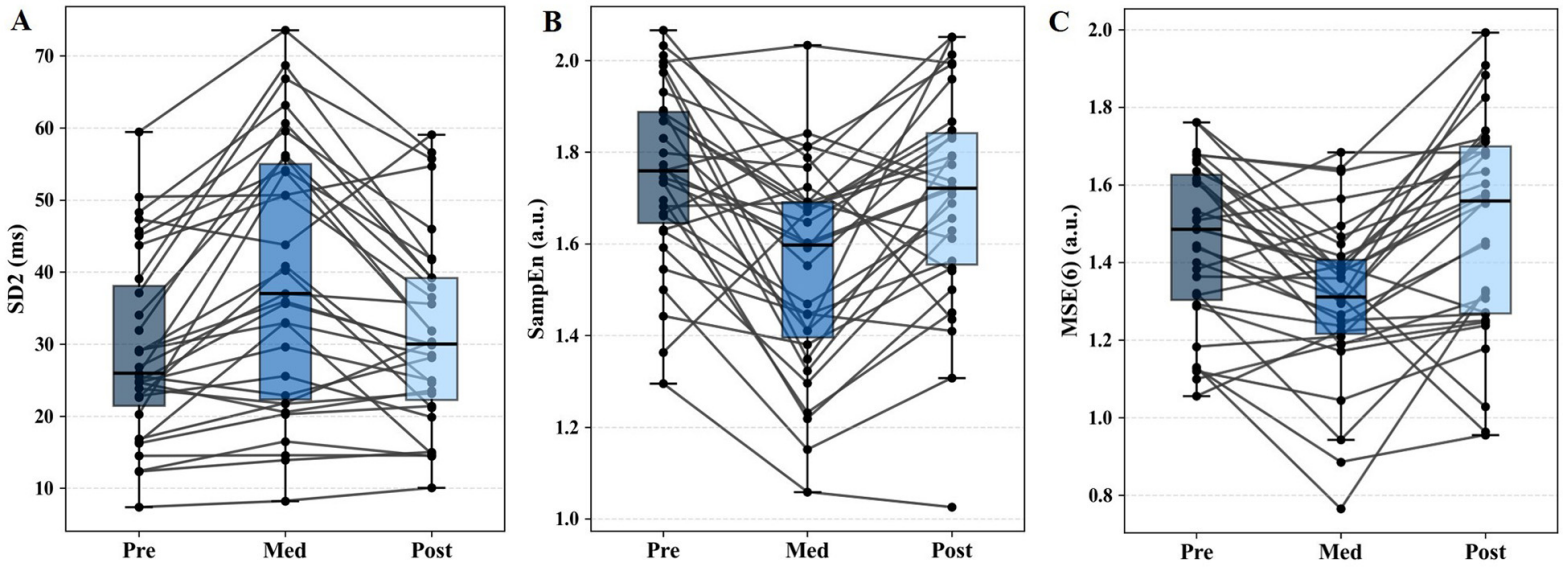
Male non-linear HRV complexity changes across the three physiological states. Boxplots of all subjects' trajectories are shown for representative non-linear indices in the male group across pre-meditation, during meditation, and post-meditation. **(A)** SD2, **(B)** SampEn, and **(C)** Multiscale entropy(6). SD2, long axis Poincar descriptor; SampEn, sample entropy.

**Figure 4:**
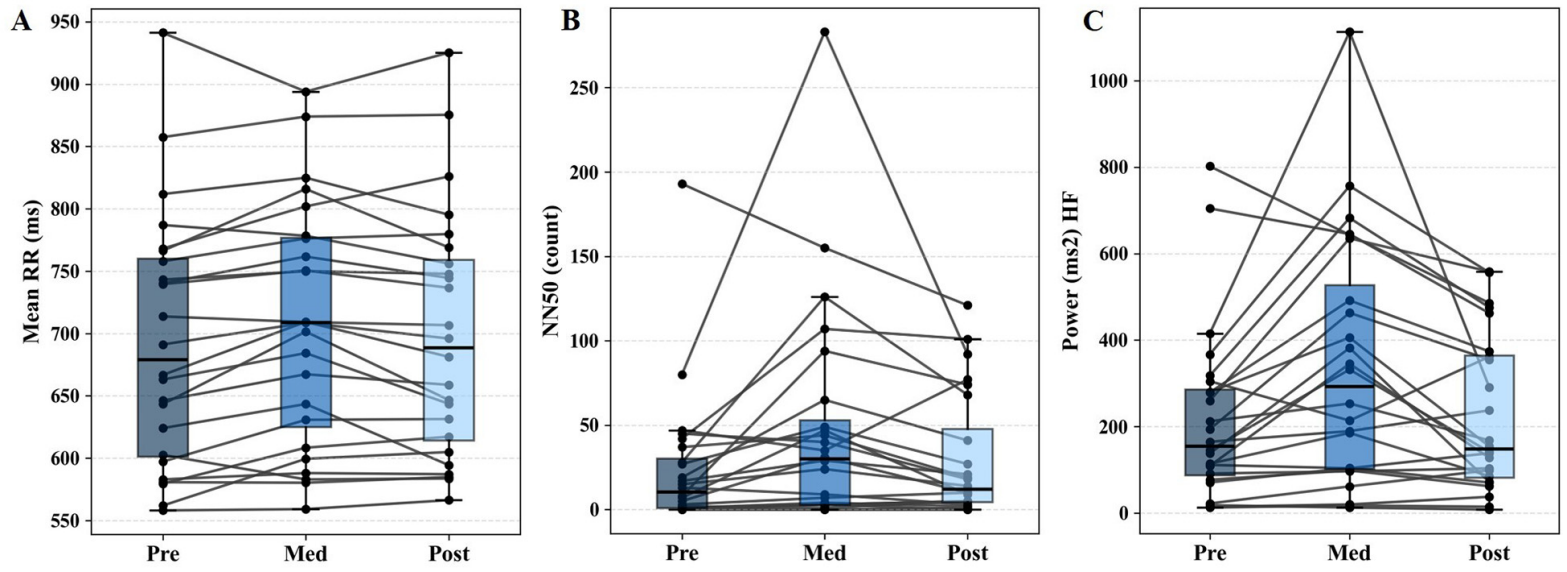
Female linear HRV and spectral changes across the three physiological states. Boxplots with participant wise trajectories are shown for representative linear and spectral indices in the female group across pre-meditation, during meditation, and post-meditation. **(A)** Mean RR **(B)** NN50 and **(C)** HF power. Mean RR, mean beat to beat interval; NN50, number of successive RR intervals differing by more than 50 ms; HF, high frequency power.

**Figure 5:**
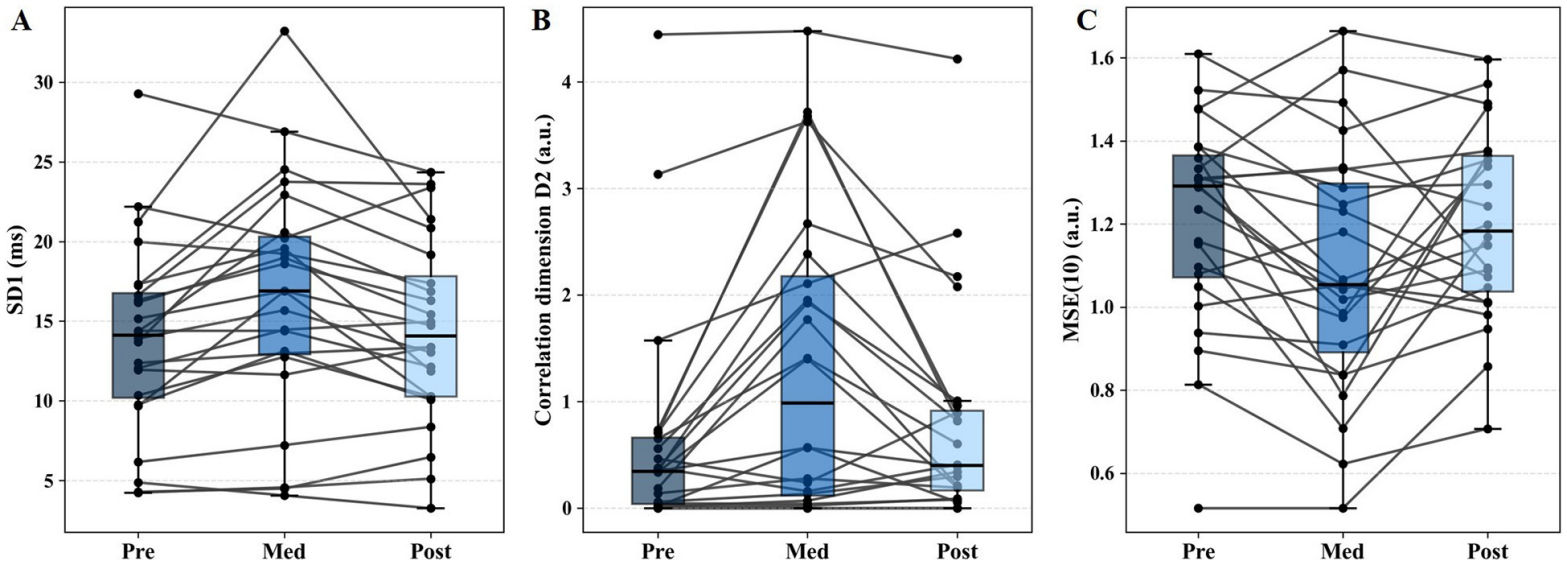
Female non-linear HRV complexity changes across the three physiological states. Boxplots with participant wise trajectories are shown for representative non-linear indices in the female group across pre meditation, during meditation, and post-meditation. **(A)** SD1 **(B)** correlation dimension D2 and **(C)** Multiscale entropy(10). SD1, short axis Poincar descriptor; D2, correlation dimension.

The Friedman test and Bonferroni adjusted *post-hoc* comparisons were applied to evaluate state-dependent changes across the pre-meditation, during-meditation, and post-meditation epochs. Linear HRV results for the male group are presented in Table 4 and for the female group in Table 5, whereas non-linear HRV results are presented in Table 6 for males and Table 7 for females. In the male group, significant time-domain changes were observed for SDNN, SD HR, Min HR, RMSSD, HRV triangular index, and TINN. Mean RR and Mean HR were not significant in males (both Friedman *p* = 0.405), and NN50 showed a non-significant trend (*p* = 0.069). SD HR and Min HR were retained as descriptive summaries of HR dispersion and the lower bound of achieved HR across each epoch. The Mean HR for females has a p value of 0.013. In the female group, significant changes were observed for Mean RR, SDNN, Mean HR, SD HR, and NN50, whereas RMSSD showed a nominal effect (Friedman *p* = 0.0175). Representative boxplots are shown in Figures 2–5.

**Table 4:** Statistical assessment of linear HRV features in the male group across pre meditation, during meditation, and post meditation states.

HRV	Friedman	Bonferroni *post-hoc* test			Median (25%–75%) IQR		
Metrics	Test	Test 1	Test 2	Test 3	Pre	Med	Post
SDNN	0.0006	0.0004	0.38	0.066	20.8 (16.8–32.2)	28.88 (17.6–43.2)	24.63 (16.6–32.9)
STD HR	0.0038	0.002	0.612	0.1264	2.32 (1.9–2.9)	3.48 (2.3–4.0)	2.41 (1.9–3.2)
Min HR	0.0093	0.008	0.044	1	64.2 (64.0–79.9)	69.46 (60.0–75.2)	70.83 (63.7–80.8)
RMSSD	0.0087	0.006	0.751	0.1703	19.54 (12.5–34.7)	26.09 (14.7–55.1)	23.33 (14.0–40.9)
HRV TI	0.0007	0.002	0.0925	0.2262	6.03 (4.7–8.5)	7.48 (5.7–9.7)	6.51 (4.5–8.7)
TINN	0.0142	0.0183	1	0.0769	127 (91–193.5)	159 (102–235.2)	141 (90–180.7)
Power (ms^2^) LF	0.0012	0.0008	0.37	0.106	210.57 (94.3–441.0)	450.44 (155.4–921.6)	249.49 (98.6–572.6)
Power (ms^2^) HF	0.0041	0.003	0.753	0.1061	189.02 (56.8–387.7)	320.58 (96.5–544.4)	215.76 (65.8–358.9)
Respiration frequency (Hz)	0.0005	0.0005	1	0.0158	0.3 (0.27 - 0.35)	0.28 (0.24–0.31)	0.3 (0.27–0.33)

Values are reported as median (25th–75th percentile). *Post-hoc* note: Test 1 = Pre vs. During; Test 2 = Pre vs. Post; Test 3 = During vs. Post. HRV TI, HRV triangular index; TINN, Triangular Interpolation of NN interval histogram.

**Table 5:** Statistical assessment of linear HRV features in the female group across pre meditation, during meditation, and post meditation states.

HRV	Friedman	Bonferroni *post-hoc* test			Median (25%–75%) IQR		
Metrics	Test	Test 1	Test 2	Test 3	Pre	Med	Post
Mean RR	0.013	0.01	0.18	0.93	678.9 (599–762)	708.91 (619–777)	688.61 (611–762)
SDNN	0.003	0.002	0.06	0.93	23.34 (16–28)	29.29 (18–40)	24.71 (19–31)
Min HR	0.013	0.01	0.18	0.936	88.40 (78.7–100)	84.64 (77–96)	87.14 (78–98)
STD HR	0.007	0.007	0.09	1	3.08 (1.7–4.1)	3.32 (2.04–5.17)	3.50 (1.88–4.46)
RMSSD	0.017*	0.018	1	0.129	19.97 (14.2–23.9)	23.87 (18.1–28.83)	19.87 (14.52–25.83)
NN50	0.005	0.003	0.321	0.321	10.5 (1.0–32.5)	30 (3.0–57.0)	12 (4.0–54.5)
Power (ms^2^) LF	0.0008	0.002	0.004	1	189.31 (101.2–393)	327.57 (105–820)	309.45 (175–594)
Power (ms^2^) HF	0.004	0.007	1	0.028	154.95 (84.26–292.4)	292.71 (101.1–563.7)	147.77 (78.7–367.8)
LF/HF	0.010	1	0.042	0.018	1.28 (0.65 - 2.29)	1.41 (0.68–2.98)	1.44 (1.04–3.73)
Respiration frequency (Hz)	0.002	0.004	1	0.013	0.32 (0.30 –0.35)	0.3 (0.25–0.32)	0.32 (0.28–0.37)

Values are reported as median (25th–75th percentile). *Post-hoc* note: Test 1 = Pre vs. During; Test 2 = Pre vs. Post; Test 3 = During vs. Post. ^*^Indicates nominal Friedman-test significance at *p* ≤ 0.05.

**Table 6:** Statistical assessment of non-linear HRV features in the male group across pre-meditation, during meditation, and post meditation states.

HRV	Friedman	Bonferroni *post-hoc* test			Median (25%–75%) IQR		
Metrics	Test	Test 1	Test 2	Test 3	Pre	Med	Post
SD2	0.0008	0.0004	0.22	0.0332	25.96 (20.8–38.5)	36.99 (22.0–55.4)	29.99 (21.8–39.2)
SD2/SD1	0.002	0.002	1	0.047	1.86 (1.5–2.4)	2.21 (1.8–2.7)	1.74 (1.6–2.5)
ApEn	0.0002	0.0003	1	0.001	1.43 (1.4–1.48)	1.37 (1.2–1.4)	1.42 (1.3–1.47)
SampEn	0.0002	0.0001	0.2962	0.0474	1.76 (1.6–1.8)	1.60 (1.3–1.6)	1.72 (1.5–1.8)
α_1_	0.013	0.010	0.612	0.296	1.04 (0.89–1.21)	1.19 (0.99–1.34)	1.11 (0.92–1.30)
α_2_	0.0017	0.015	1	0.002	0.43 (0.3–0.5)	0.37 (0.2–0.5)	0.48 (0.3–0.5)
CD , D2	0.039*	0.042	1	0.212	0.26 (0.05–1.0)	0.57 (0.09–3.4)	0.37 (0.07–1.08)
RP Lmean	0.020*	0.015	0.487	0.487	9.26 (7.9–10.4)	11.10 (9.3–12.1)	9.60 (8.4–11.0)
RP DET	0.013	0.015	0.092	1	96.83 (95.8–98.5)	98.15 (97.0–98.6)	97.28 (96.3–98.2)
RP ShanEn	0.036*	0.033	0.296	1	3.01 (2.8–3.1)	3.20 (3.0–3.3)	3.04 (2.9–3.2)
MSE(1)	0.0002	0.0001	0.29	0.04	1.76 (1.6–1.8)	1.60 (1.3–1.7)	1.72 (1.5–1.8)
MSE(2)	0.0027	0.001	0.17	0.38	1.82 (1.7–1.88)	1.73 (1.6–1.8)	1.80 (1.6–1.85)
MSE(6)	0.0001	0.010	0.226	0.007	1.49 (1.3–1.6)	1.31 (1.2–1.4)	1.56 (1.2–1.7)
MSE(9)	0.002	0.001	0.75	0.06	1.22 (1.05–1.4)	1.07 (0.9–1.2)	1.24 (0.9–1.4)
MSE(10)	0.004	0.006	1	0.022	1.13 (0.8–1.3)	0.95 (0.83–1.1)	1.12 (0.87–1.28)
MSE(12)	0.008	0.0069	0.75	0.17	1.0 (0.77–1.1)	0.84 (0.68–1.01)	0.90 (0.70–1.1)
MSE(13)	0.03*	1	0.066	0.066	0.8 (0.57–1.0)	0.79 (0.67–0.95)	0.85 (0.62–1.1)
MSE(15)	0.008	1	0.033	0.015	0.70 (0.51–0.88)	0.70 (0.50–0.86)	0.80 (0.5–0.96)

Values are reported as median (25th–75th percentile). *Post-hoc* note: Test 1 = Pre vs. During; Test 2 = Pre vs. Post; Test 3 = During vs. Post.

RP Lmean, Recurrence Plot Mean diagonal line length; RP DET, Recurrence Plot Determinism; RP ShanEn, Recurrence Plot Shannon Entropy; SD2/SD1, Ratio of long-term to short-term variability. ^*^Indicates nominal Friedman-test significance at *p* ≤ 0.05.

**Table 7:** Statistical assessment of non-linear HRV features in the female group across pre meditation, during meditation, and post meditation states.

HRV	Friedman	Bonferroni *post-hoc* test			Median (25%–75%) IQR		
Metrics	Test	Test 1	Test 2	Test 3	Pre	Med	Post
SD1	0.011	0.011	1	0.10	14.13 (10.06–16.9)	16.89 (12.8–20.4)	14.06 (10.2–18.27)
SD2	0.003	0.002	0.28	0.28	29.43 (19.65–36.8)	36.62 (22.6–51.2)	32.33 (24.3–40.2)
ApEn	0.001	0.0014	0.04	0.82	1.44 (1.31–1.49)	1.40 (1.2–1.4)	1.41 (1.31–1.48)
SampEn	0.047*	0.040	0.652	0.0652	1.72 (1.46–1.85)	1.60 (1.2–1.8)	1.60 (1.4–1.83)
α_1_	0.037*	0.331	0.033	1	1.07 (0.91–1.23)	1.18 (0.84–1.4)	1.11 (0.98–1.34)
α_2_	0.003	1	0.04	0.003	0.50(0.43–0.56)	0.43 (0.3–0.58)	0.54 (0.48–0.63)
CD, D2	0.0004	0.0008	0.005	1	0.35 (0.03–0.68)	0.99 (0.1–2.2)	0.40 (0.14–0.9)
MSE(1)	0.047*	0.04	0.65	0.65	1.72 (1.46–1.85)	1.60 (1.28–1.8)	1.60 (1.42–1.83)
MSE(5)	0.027	0.44	0.65	0.021	1.52 (1.44–1.67)	1.47 (1.23–1.58)	1.59 (1.45–1.70)
MSE(8)	0.013	0.017	1	0.073	1.36 (1.27–1.53)	1.30 (1.06–1.48)	1.40 (1.2–1.5)
MSE(10)	0.050*	0.060	1	0.209	1.29 (1.06–1.37)	1.05 (0.87–1.31)	1.18 (1.03–1.36)
MSE(11)	0.03	0.88	1	0.049	1.14 (0.94–1.31)	1.09 (0.89–1.25)	1.18 (1.06–1.32)

Values are reported as median (25th–75th percentile). *Post-hoc* note: Test 1 = Pre vs. During; Test 2 = Pre vs. Post; Test 3 = During vs. Post.

MSE, Multiscale entropy; SD1, short term variability in a Poincar plot; SD2, long term variability in a Poincaré plot; CD D2, Correlation Dimension; α_1_, Short-term fractal scaling exponent; α_2_, Long-term fractal scaling exponent. ^*^Indicates nominal Friedman-test significance at *p* ≤ 0.05.

For the male group, Figures 2, 3 illustrate boxplots of representative time, frequency and non-linear HRV indices (SDNN, LF power, respiration frequency (Hz), SD2, SampEn, and MSE (6)) across the three physiological states (pre, during and post-meditation). The corresponding female boxplots for Mean RR, NN50, HF power, SD1, correlation dimension *D*_2_ and MSE (10) are shown in Figures 4, 5, respectively, all exhibiting significant changes (*p* < 0.01). Notably, the modulation of vagal and non-linear indices was more prominent in females, consistent with the significant LF/HF ratio changes observed only in the female group.

#### 3.1.2 Non-linear parameters

Most non-linear indices showed significant modulation in both groups. In males, SD2, SD2/SD1, ApEn, SampEn,short term fluctuations (α_1_), long term fluctuations (α_2_), recurrence-plot DET and several MSE scales [MSE (1), MSE (2), MSE (6), MSE (9), MSE (10), MSE (12), MSE (15)] were significant at the 1% level, while CD D2, recurrence-plot Lmean and ShEn reached 5% significance (Table 6). In females, SD1, SD2, ApEn, α_2_, CD D2 and MSE (8) were significant at 1%, whereas SampEn, α_1_, MSE (1), MSE (5), MSE (10) and MSE (11) showed 5% significance (Table 7). The representative non-linear indices plotted in Figures 3, 5 (SD2, SampEn and MSE (6) for males; SD1, CD D2 and MSE (10) for females) reveal a consistent reduction in multiscale entropy during meditation more pronounced in males together with transient changes in long term variability and fractal complexity. HRV respiration analysis as 27 significantly modulated features in males and 22 in females, designates a more shift toward parasympathetic predominance during RM; these results are strengthened by rigorous artifact detection and rejection in Kubios HRV Scientific, and by the observation that recurrence-plot indices changed noticeably in the male group.

### 3.2 Correlation between HRV and respiration

**Figure 6:**
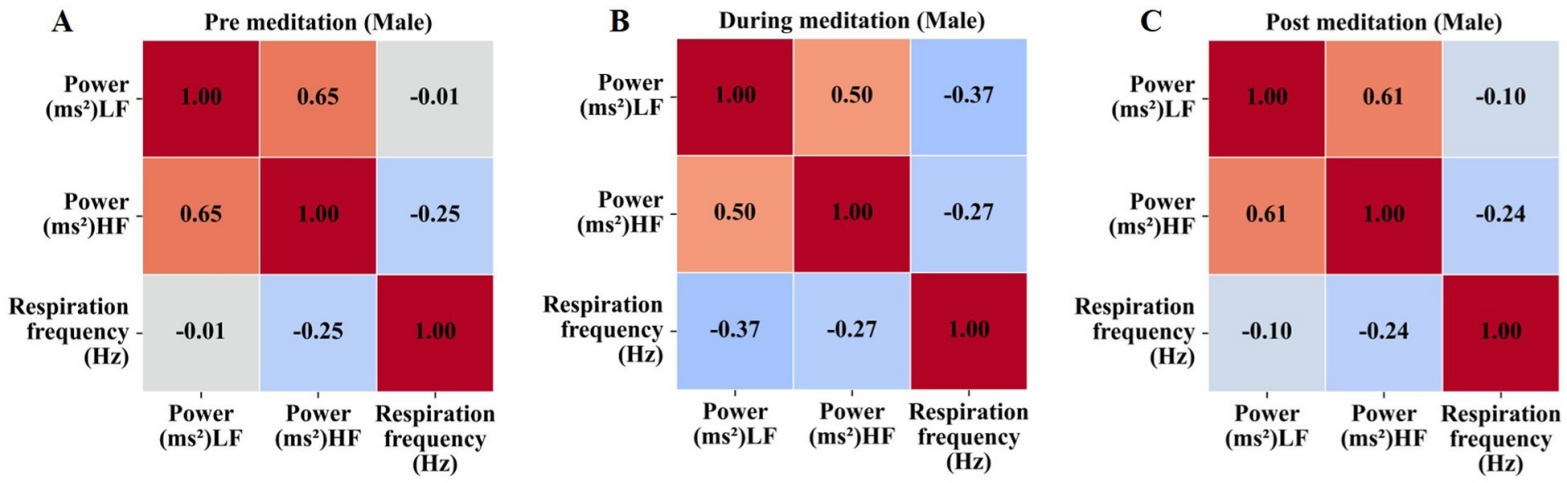
Male cardiorespiratory correlation matrices across the three physiological states. State-wise correlation matrices are shown for the male group. **(A)** Pre meditation, **(B)** during meditation, and **(C)** post-meditation. The displayed coefficients represent the associations among LF power, HF power, and respiration frequency within each state. LF, low frequency power; HF, high frequency power.

**Figure 7:**
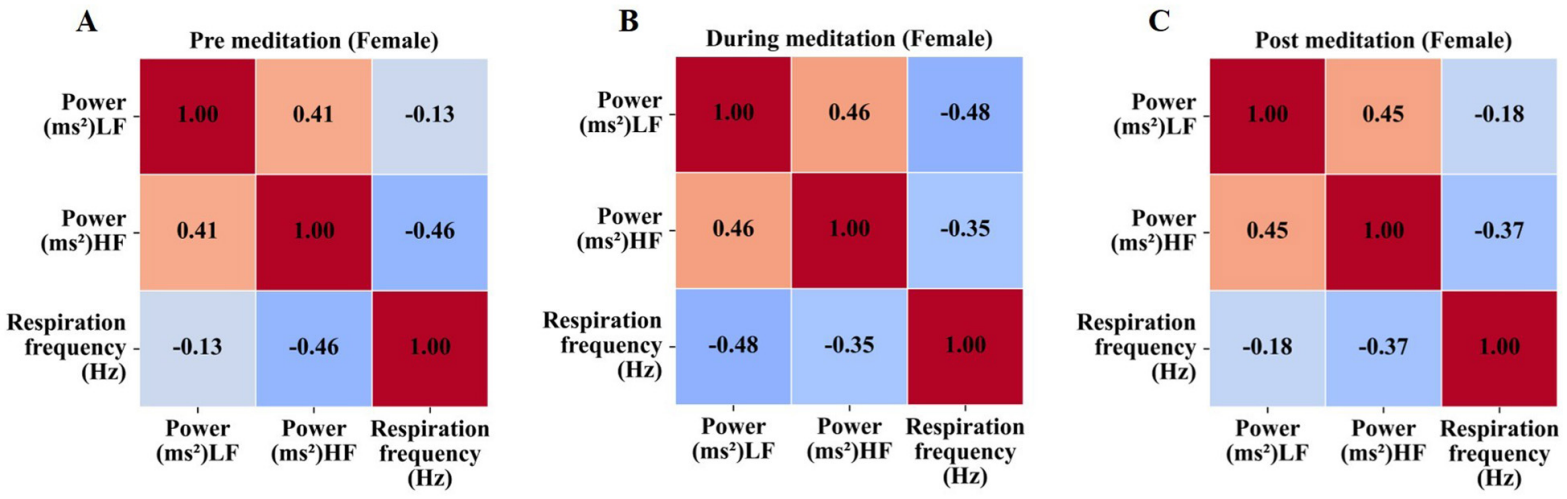
Female cardiorespiratory correlation matrices across the three physiological states. State-wise correlation matrices are shown for the female group. **(A)** Pre meditation, **(B)** during meditation, and **(C)** post-meditation. The displayed coefficients represent the associations among LF power, HF power, and respiration frequency within each state. LF, low frequency power; HF, high frequency power.

In the male group during meditation, a moderately positive correlation (*r* = 0.50) between LF power and HF power was observed, indicating that increases in LF power tended to be accompanied by increases in HF power. A moderate negative association (*r* = –0.37) between LF power and respiration frequency was also noted, implying that higher LF power was generally accompanied by lower respiration frequency. In addition, a weaker negative correlation (*r* = –0.27) between HF power and respiration suggested that increases in HF power were linked with slight reductions in respiration frequency. The associations in the male group for pre meditation, during meditative and post-meditative conditions are shown in Figure 6. For female participants, the correlation between LF power and HF power during meditation (*r* = 0.46) was found to be stronger than in the pre meditative phase (*r* = 0.41), indicating a compact alignment between these HRV components when meditation was practiced. The converse bonds between LF power and respiration, and between HF power and respiration, were also found to be more pronounced during meditation, which suggests that higher HRV power tended to coincide with slower breathing. The corresponding correlation matrices for females in the three phases are displayed in Figure 7.

## 4 Discussion

The present findings suggest that Rajyoga meditation is accompanied by an acute shift toward a calmer and more regulated physiological state. Across the sample, meditation was associated with changes in HRV and respiration in a direction broadly consistent with greater parasympathetic engagement and reduced physiological arousal. Framed in psychological rather than technical terms, the pattern is more consistent with calm regulation than with stress-related activation. At the same time, any differences between male and female subgroups should be treated as descriptive and exploratory, not as definitive evidence of sex specific effects.

### 4.1 HRV dynamics and the physiological effects of RM

In both groups, meditation was associated with changes in HRV indices and respiration frequency in a direction consistent with enhanced parasympathetic engagement. In males, significant changes were observed in several time-domain and non-linear indices, whereas in females clearer modulation was observed in Mean RR, Mean HR, NN50, and selected spectral measures. These subgroup differences should be interpreted cautiously because the sex stratified analyses were exploratory rather than interaction-powered. LF power is frequently associated with both SNS and PNS (Pomeranz et al., 1985). The increase may indicate that autonomic balance has been stabilized, potentially due to the improved functioning of the parasympathetic system during meditation. During meditation, both genders' poincare plot SD1, SD2, and ratio of SD2/SD1 increase (Soni and Muniyandi, 2019), suggesting increased short term variability in HR and increased parasympathetic regulation. An improvement in autonomic control is shown by an increase in SD2, which reflects increased long term variability and overall HRV. Meditation lowers ApEn and SampEn, indicating more regular HR dynamics. Entropy values decrease with meditation, suggesting a more stable autonomic state. Stress reduction and emotional regulation benefit from this stability, which indicates a calmer and less varied autonomic response. Meditation practice may decrease ApEn and SampEn by relaxing and lowering autonomic tension, resulting in a more stable HR. The observed rise in DFA α_1_ may be linked to a lowering in immediate cardiovascular stress and a more harmonized autonomic reaction during meditation, hence promoting cardiovascular wellbeing and adaptability. α_2_ illustrates HRV's long term scaling characteristic. A decline in α_2_ may indicate decreased variability in HR over longer time spans and a reduction in long term fractal correlations. Among all non-linear attributes, significant differences in recurrence quantification analysis (RQA) were observed in the male class, whereas no significant differences were found in the female class. Furthermore, MSE measures displayed a higher rate of significant differences in the male compared to the female group.

### 4.2 Physiological implications and real world relevance

The observed increases in vagally mediated and variability related HRV indices during meditation may be interpreted as reflecting a more regulated autonomic state, with greater parasympathetic engagement and improved short-term physiological flexibility. In psychophysiological literature people with higher HRV tend, on average, to show better emotion regulation, stronger executive control, and better adaptation to stress (Laborde et al., 2017; Holzman and Bridgett, 2017). Accordingly, the present pattern of increased HRV during Rajyoga meditation may be translated as a physiological state more consistent with calm engagement than with stress related arousal.

The concurrent reduction in respiration frequency during meditation may also be interpreted in practical terms. Slower and more regular breathing has been associated with autonomic stabilization, improved cardiorespiratory coordination, and greater psychological flexibility (Zaccaro et al., 2018). Therefore, the combined HRV and respiration findings suggest that Rajyoga meditation may acutely support a state of reduced physiological strain, better emotional regulation, and more stable autonomic control. Because the post-meditation state showed only partial return toward baseline, the findings may further indicate that some of these regulatory effects persist for a short period after meditation has been discontinued.

### 4.3 HRV and respiration correlation

Cardiorespiratory coupling was interpreted through the state-wise correlations among LF power, HF power, and respiration frequency. This analytical framework is widely used because cardiac and respiratory rhythms are physiologically coupled and can be examined jointly to characterize autonomic regulation (Dick et al., 2014). LF and HF powers, together with ECG-derived respiration, were obtained using Kubios HRV (Tarvainen et al., 2014). At the same time, LF/HF related interpretation was kept cautious, because the LF/HF ratio should not be treated as a direct quantitative surrogate of sympathovagal balance (Billman, 2013).

### 4.4 Comparative analysis

Prior studies have largely concentrated on two states: meditation and premeditation. To provide a more comprehensive knowledge of the effects of meditation on the ANS, this study expands the analysis to include the post-meditation phase. Earlier studies on RM have not considered the within subject variations in HRV response of three meditative states. Studies such as Soni and Muniyandi (2019) generalized the effects of meditation to the entire population without considering the influence of the correlation of meditative states. In contrast, this study investigates male groups and female groups individually, revealing notable within subject differences in HRV properties. Although earlier work, such as Goshvarpour et al. (2017) employed entropy measurements for HRV analysis, generally maintained a single scale for multiscale entropy. Using various scales of MSE, this work is the first to provide a more nuanced understanding of the complexity of HRV signals in non-linearity. Such features enable a more profound understanding of the mechanisms of autonomic control during meditation. Most previous work has not considered the correlation between respiration and HRV. Previous research (Kuppusamy et al., 2020) has only examined HRV parameters and not how respiration affects these metrics. This work closes this knowledge gap by investigating the relationship between HRV (more specifically, LF and HF power) and respiration, providing important new information about how the circulatory and respiratory systems interact during meditation. Table 8 presents a comparison of the proposed work with recent studies.

**Table 8:** Comparison of the proposed work with recent studies.

Author and years	Modality	States	HRV-respiration correlation	Results
Deepeshwar and Budhi (2022)	Slow yoga breathing	Before and after	No	Immediate autonomic changes
Natarajan (2023)	Mindfulness	Rest and meditation	No	Introduces ABI as alternative meditation metric
Nasrolahzadeh et al. (2023)	Chi Meditation	Pre and during	No	*P* < 0.05
Chhabra et al. (2024)	Sudarshan Kriya Yoga	Session protocol	Cardio-respiratory synchronization	Shows strengthened cardiovascular–respiratory synchronization
Jha et al. (2025)	Rajyoga	Pre -Post	No	RM improved parasympathetic indices not within-session
Proposed study	Rajyoga	Pre during post	Yes	Pre-during-post non-linear features (MSE1–20) and explicit HRV–respiration coupling rigorous within-subject design

### 4.5 Limitations and future directions

This study has several limitations that could be acknowledged in future study. The cohort was modest in size and limited to trained Rajyoga practitioners, which may constrain generalizability across populations and meditation traditions. Future studies should include larger samples and multiple meditation types to determine the robustness and specificity of these findings. In addition, our interpretation of altered consciousness and brain-body regulation was based on autonomic and respiratory measures derived from single lead ECG, without direct assessment of neural activity. Multimodal studies combining ECG with electroencephalogram (EEG) and other peripheral indices such as galvanic skin response will be important for defining the neural basis of the observed physiological changes. Mechanistically, computational modeling (Panda et al., 2023, 2022) may further clarify how meditation modulates interactions among respiration, autonomic regulation, and brain state, and how these changes relate to wellbeing. Finally, because this study was conducted in healthy practitioners, the clinical relevance of these markers remains uncertain and should be tested in neuropsychiatric and other patient populations.

## 5 Conclusion

Rajyoga meditation modulates HRV and cardiorespiratory coupling. Our multiscale entropy analyses indicated broader parasympathetic engagement in female participants, highlighting potential sex specific patterns in autonomic adaptability. Across both groups, meditation increased short term HRV stability while reducing long term variability, suggesting improved autonomic regulation and cardiovascular resilience. Strengthened negative correlations between respiration and low frequency HRV power further support parasympathetic activation. These findings support Rajyoga meditation as a modulator of autonomic complexity, offering insight into the embodied signatures of contemplative consciousness.

## Data Availability

The raw data supporting the conclusions of this article will be made available by the authors, without undue reservation.
